# Supplementary material for: A high-resolution mRNA expression time course of embryonic development in zebrafish
Source: eLife. 2017 Nov 16;6:e30860. doi: 10.7554/eLife.30860 (PMC5690287; doi:10.7554/eLife.30860)
Supplement: Supplementary file 6. [file elife-30860-supp6.zip › biolayout-clusters-files/Cluster214.html]

Cluster214


## Cluster214: Detail

### Go to GO detail

## ZFA

| ZFA ID | Description | Annotated | Expected | Observed | Fold Enrichment | Adjusted p-value | Genes | Ensembl IDs |
| --- | --- | --- | --- | --- | --- | --- | --- | --- |
| ZFA:0000088 | YSL | 581 | 0.33 | 6 | 18 | 0.0018 | atic hmox1a fahd2a apoa4a dao.1 zgc:136410 | ENSDARG00000016706 ENSDARG00000027529 ENSDARG00000103759 ENSDARG00000101160 ENSDARG00000035602 ENSDARG00000102241 |
